# Palytoxin and Analogs: Biological and Ecological Effects

**DOI:** 10.3390/md8072021

**Published:** 2010-06-30

**Authors:** Vítor Ramos, Vítor Vasconcelos

**Affiliations:** 1 Marine and Environmental Research Center–CIIMAR/CIMAR, Porto University, Rua dos Bragas, 289, 4050-123 Porto, Portugal; E-Mail: bioramolas@hotmail.com; 2 Faculty of Sciences, Porto University, Rua do Campo Alegre, 4169-007 Porto, Portugal

**Keywords:** marine toxins, palytoxin, ostreocin, mascarenotoxin, ovatoxin, Palythoa, Ostreopsis

## Abstract

Palytoxin (PTX) is a potent marine toxin that was originally found in soft corals from tropical areas of the Pacific Ocean. Soon after, its occurrence was observed in numerous other marine organisms from the same ecological region. More recently, several analogs of PTX were discovered, remarkably all from species of the dinoflagellate genus *Ostreopsis*. Since these dinoflagellates are also found in other tropical and even in temperate regions, the formerly unsuspected broad distribution of these toxins was revealed. Toxicological studies with these compounds shows repeatedly low LD_50_ values in different mammals, revealing an acute toxic effect on several organs, as demonstrated by different routes of exposure. Bioassays tested for some marine invertebrates and evidences from environmental populations exposed to the toxins also give indications of the high impact that these compounds may have on natural food webs. The recognition of its wide distribution coupled with the poisoning effects that these toxins can have on animals and especially on humans have concerned the scientific community. In this paper, we review the current knowledge on the effects of PTX and its analogs on different organisms, exposing the impact that these toxins may have in coastal ecosystems.

## 1. Palytoxin Chemical Properties and Producing Organisms

**Figure 1:**
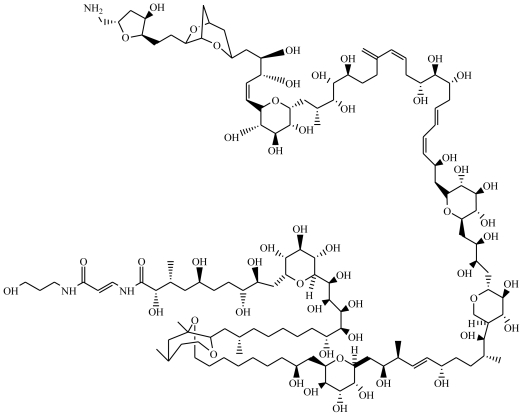
Structure of palytoxin (C_129_H_227_N_3_O_52_, molecular weight 2652.17 g mol^−1^).

Palytoxin (PTX) is a large, very complex molecule (Figure 1) with a long polyhydroxylated and partially unsaturated aliphatic backbone, containing 64 chiral centers [1]. This latter feature, coupled with the presence of eight double bonds that are able to exhibit cis/trans-isomerism means that PTX can have more than 10^21^ stereoisomers [2]. PTX was originally isolated in Hawaii from the tropical soft coral *Palythoa* sp., a zoanthid [3]. In 1981, its unique chemical structure was elucidated independently by two groups [4,5] while in 1994 it was fully synthesized for the first time [6]. Since then, chemical structures of some of the analogs of PTX were also achieved, e.g., ostreocin [7,8], mascarenotoxins [9,10] and ovatoxin [11]. Notably, all are produced by dinoflagellates from the genus *Ostreopsis*. For a review of the chemistry of PTX and its analogs see [12].

The molecular weights of PTX range from 2659 to 2680 Da, depending on the *Palythoa* species from which it is obtained [13]. Ostreocin-D is 2635 Da [7,8], mascarenotoxin-A and mascarenotoxin-B range between 2500 and 2535 Da [12], while ovatoxin is 2648 Da [11]. PTX has both lipophilic and hydrophilic regions and is referred to as a super-carbon-chain compound, since it has the longest chain of continuous carbon atoms in any known natural product [14,15]. It is heat-stable, not inactivated by boiling, and is stable in neutral aqueous solutions for prolonged periods, however a rapid decomposition occurs under acid or alkaline conditions, leading to loss of its toxicity [2].

A vast effort has been devoted to determining the mechanism of action of PTX (see [16] and [17] for reviews). At the sub-cellular level, the widely accepted molecular action of PTX is blockage of the Na^+^/K^+^-ATPase channel [17–19], a transmembrane protein (also known as a sodium pump) that transports three Na^+^ out of the cell and two K^+^ in, using ATP hydrolysis as the driving force. The electrochemical gradient generated by the sodium pump is essential for the maintenance of cell homeostasis [16]. PTX seems to bind to the extracellular part of the Na^+^/K^+^-ATPase, and thereby inhibits the active transport of Na^+^ and K^+^ across the cell membrane by transforming the pump into a non-specific permanently open ion channel. The membrane depolarization generated and the massive increase of Ca^2+^ in the cytosol [20] interferes with some vital functions of cells. An altered concentration of intracellular cations, in particular a calcium increase, is generally associated with cell death [21]. In addition to the stimulating release of K^+^ from various cell types and the depolarization of all excitable tissue investigated [16], PTX causes a wide spectrum of secondary pharmacological actions. These include violent contraction of skeletal, smooth and cardiac muscles, cardiovascular effects, hemolysis, histamine, prostaglandin and norepinephrine release, platelet aggregation, bone resorption and inhibition of sperm motility [16,17,22–25].

Other studies highlight the cytoskeleton as an early target for the toxic effects of PTX and its analog ostreocin-D on intestinal [26] and neuroblastoma cells [18,27]. It was demonstrated that the signaling cascade triggered by PTX and ostreocin-D leads to actin filament system distortion [28], while pointing out that factors other than Ca^2+^ influx must be involved in how these toxins effect the cellular actin cytoskeleton. The studies revealed that ostreocin-D has a behavior very similar to PTX, and also indicate that both toxins share the same targets. Nevertheless, the small structural differences between PTX and ostreocin-D cause a significant reduction in cytotoxicity and hemolytic potency of the latter [12]. PTX has also been recognized as a potent tumor promoter [17,29], but with the particularity that it does not activate protein kinase C, the receptor for the prototypical skin tumor promoter 12-*O*-tetradecanoylphorbol-13-acetate (TPA) [30]. Hence, the understanding of palytoxin action can reveal new aspects of tumor promotion, which can be used for anticancer purposes—something that had been suggested in the 1990s but forgotten in the meantime [29].

Although primarily found on *Palythoa* spp., PTX was also detected in organisms living in close association with the colonial zoanthids [31]. Moreover, PTX and analogs were extracted from many other marine organisms (see [16] for an extensive list) including primary producers such as the red alga *Chondria crispus* [32] and the benthic dinoflagellates *Ostreopsis* spp. [7]. In addition, bacteria associated with preceding organisms have also been studied as a possible source for the production of this nonproteinaceous toxin. This is supported by the fact that PTX hemolytic activity was detected in extracts of bacteria such as *Pseudomonas* [33], *Brevibacterium, Acinetobacter* and from the *Bacillus cereus* group [34]. It was also found that *Vibrio* sp. and *Aeromonas* sp. are able to produce compounds antigenically related to PTX [35]. Thereby, the presence of PTX and analogs in this myriad of marine organisms once can suggest a bacterial origin for the toxin production [34,36].

## 2. Toxicity of PTX and Its Analogs in Higher Animals

PTX is one of the most toxic non-peptide substances known [37], showing remarkable biological activity even at very low concentration [3]. This toxin and its analogs have become a global concern due to the poison’s effects on animals and especially on humans. Moreover, the recognition that they have a wide geographical distribution and can be found in a variety of seafood has reinforced this concern [38,39].

### 2.1. Toxicity in Humans: Reports, Symptoms and Routes of Exposure

Human fatalities due to consumption of seafood suspected to be contaminated with PTX were reported in the Philippines, after consumption of the crab *Demania reynaudii* [40], and in Madagascar following consumption of the sardine *Herklotsichthys quadrimaculatus* [41]. In fact, PTX is now suggested to be the cause of clupeotoxism—a poorly understood toxic syndrome associated with the consumption of clupeoid fish, such as sardines, herrings and anchovies, and characterized by a high mortality rate [38,41]. Near fatal cases took place after consumption of a smoked fish (*Decapterus macrosoma*) in Hawaii [42] and after groupers (*Epinephelus* sp.) or blue humphead parrotfish (*Scarus ovifrons*) were eaten in Japan [43,44]. The most commonly reported complications of PTX poisoning appears to be rhabdomyolysis [42–44], a syndrome injuring skeletal muscle, causing muscle breakdown, and leakage of large quantities of intracellular (myocyte) contents into blood plasma [38]. Other symptoms associated with PTX poisoning in humans are characterized by a bitter/metallic taste, abdominal cramps, nausea, vomiting, diarrhea, paresthesia, bradycardia, renal failure, cyanosis and respiratory distress [40,42,43]. The latter two precede death in fatal cases [29].

Recent cases of human intoxication by PTX have shown different contours. In Germany [45] and in the USA [38], poisoning occurred through dermal absorption, after the victims touched zoanthid corals present in their home aquariums. Moreover, a case of human exposure to PTX via inhalation was also reported regarding a patient who attempted to kill a *Palythoa* coral in his aquarium [46]. Many other anecdotal evidences of intoxications related with aquarium zoanthids can be found in online marine aquarium forums [38]. Respiratory illness has also occurred when people were exposed to *Ostreopsis ovata* bloom aerosols during recreational or working activities, in Italy [47]. Symptoms caused by ovatoxin-a from aerosols include fever associated to serious respiratory disturbs, such as bronchoconstriction, mild dyspnea, and wheezes, while conjunctivitis was observed in some cases [11,47,48]. These reports highlight the existence of new routes of exposure to PTX and its analogs while it alerts for the risks experienced by aquarium hobbyists by keeping zoanthid in these artificial ecosystems. For a detailed review of human health risks, see [38].

### 2.2. Toxicity in Other Mammals: Lethal and Sub-Lethal Effects

PTX is hemorrhagic to mammals and has significant effects on cardiovascular, kidney, gastrointestinal and respiratory systems [49]. This toxin has been known to be extremely lethal to animals by intraperitoneal or intravenous administration, since its discovery [3,49]. It can be detected and quantitatively measured by the use of biological assays, although chemical analytical methods are necessary to confirm its presence in natural samples [50]. In a recent review about current assays for marine toxins, it was recognized that the mouse test is the most widely used for sample toxicity detection, and even the reference method for marine toxins in most countries [37]. Nevertheless, for palytoxin and analogs, this bioassay currently in-use is not yet fully developed [37,51]. For instance, data regarding mouse assays for PTX varies in the literature, since authors use different definitions to “mouse unit”, detection limits, LD_50_ values (which ranges between 0.150 and 0.720 μg/kg), and observation time of mice (from 4 to 48 h) [51]. To reach to a consensus, Riobó *et al*. [51] deeply analyzed and described the proper employment of this bioassay for PTX, since it could constitute the reference for other methods, according to the authors.

The earliest study using animal models for PTX toxicity showed highly lethal effects of this substance in several species [49], although this work was done with semi-purified material [38]. In the first attempts to determine PTX toxicity, the crude ethanol extracts of *Palythoa toxica* proved to be so toxic that an accurate LD_50_ was difficult to determine [52]. A summary of toxic effects and LD_50_ of PTX for various exposure routes, as well as for several animal tested, can be found in [29] and [38]. In a similar way, a more complete toxicity assessment for PTX analogs from *Ostreopsis* spp. can be found in [52]. For comparative purpose, the LD_50_ values of these toxins observed 24 hours after exposure in the two most common test animals are summarized in Table 1.

The intravenous LD_50_ for PTX in rats and mice is 0.089 and 0.045 μg/kg, respectively (Table 1). Toxicity values obtained by this route in other mammals—rabbit, dog, monkey, and guinea pig—ranged between 0.025 and 0.45 μg/kg, being the former the most sensitive animal model tested so far [16,38]. Symptoms at high doses include ataxia, convulsions, dyspnea, and subsequently death within minutes [29]. In this case, the rapid death is attributed to heart failure. In contrast, at doses close to the LD_50_, death occurs 8–10 h after administration, and cardiac activity still persists even after respiratory arrest [29].

Several studies [38,49] point to similar LD_50_ values for PTX in mice either by intraperitoneal (0.4–0.72 μg/kg, variation due to different sources of PTX) or by intravenous (0.15–0.74 μg/kg) administration. Corroborating these findings, the 24 h LD_50_ value for PTX in the mouse bioassay by intraperitoneal injection was recently [51] established to be 0.295 μg/kg (see Table 1). In rats, the acute toxicity by intraperitoneal administration is much lower than the intravenous LD_50_ (0.63 μg/kg *vs.* 0.089 μg/kg, respectively). Regarding the PTX analogs, ostreocin-D injected by intraperitoneal route has a LD_50_ of 0.75 μg/kg in mouse, a value similar to PTX, while mascarenotoxin-A from a crude extract of *Ostreopsis mascarenensis* has showed much lower toxicity, presenting a LD_50_ value of 900 μg/kg [7,9,12]. Despite the reported [12] mouse lethality via intraperitoneal injection of ostreotoxin-1 and -3 produced by *O. lenticularis*, the classification of these compounds as PTX analogs is still unclear. This is because, so far, analytical methods for these molecules are still lacking [29,54]. Some studies [51,53,55] showed that the symptoms are quite consistent whether the intoxication is achieved by purified PTX or by analogs derived from crude extracts of *Ostreopsis* spp. or from toxic fish and crabs: uncoordinated movement and paralysis are early observations; dyspnea, cyanosis and exophthalmus precede death, while convulsions and diarrhea have been described in some cases. Riobó *et al.* [51] observed that at dose levels close to the LD_50_, death may occur up to 48 h after toxin administration by intraperitoneal route. The characteristic initial symptoms of mice intoxication (within 15 min after administration) were stretching of hind limbs, lower backs and concave curvature of the spinal column, whether the mice died or stayed alive. The authors state that these early symptoms are sufficiently distinctive for allowing the identification of the presence of PTX regardless the presence of other toxins in the sample.

Although the risk associated with ingestion of contaminated seafood is well recognized [38], PTX is much less toxic orally than by parenteral administration aforementioned. Results from the few existing studies (Table 1) reports an oral LD_50_ of >40 μg/kg in rats and 510 μg/kg in mice, by intragastric injection [29,49]. Recently it was shown that toxin administration by gavage in mice did not produce death at the maximum dosage of 200 μ/kg of PTX and 300 μg/kg of ostreocin-D [56]. Nevertheless, another recent study showed lethality by acute oral administration of PTX [53], despite these values were three orders of magnitude lower than that observed after intraperitoneal injection. The deaths started at 600 μg/kg and LD_50_ was established at 767 μg/kg, a result somewhat comparable to that presented above. At lethal doses, the symptoms are similar to those observed after intraperitoneal administration (jumping, paralysis of the hind limbs and respiratory distress), suggesting that skeletal muscles, including the respiratory musculature, may be primary targets of PTX. These findings are supported by additional ultrastructural and hematoclinical data [53]. At sub-lethal doses of PTX and ostreocin-D administrations, weak erosion in the stomach caused by gastric juice secretion is observed, and also light injuries to the small intestine, lung and kidney [56]. Both toxins also induced organ injuries after 24 h when dosed by sublingual administration at about 200 μg/kg. The injuries became fatal when the PTX dose was given twice or three-times [56]. Controversially, evaluations for NOEL and LOEL values for acute oral administration of PTX in mice were estimated to be 300 μg/kg [53] and 200 μg/kg [56], respectively.

By intra-tracheal route, PTX caused mice mortality with doses above 2 μg/kg in 2 h and rats death at 5~7.5 μg/kg, with paralytic symptoms [56]. Values obtained earlier [49] in rats indicates a 24 h LD_50_ of 0.36 μg/kg (Table 1). Ostreocin-D showed to be less potent than PTX, since mice died at 13 μg/kg after 1 h or 11 μg/kg after 6 h [56]. At sublethal dose (1 μg/kg), mice and rats were unable to walk for 1–2 h but recovered thereafter. After 24 h, surviving animals appeared to be in normal condition, but with multiple organ injuries (lung, gastro-intestines and kidney). The study [56] also indicates that the two toxins administered via the trachea move to the lung and further to other organs. Though slight differences could be seen, both toxins were very toxic by this route, which is a model for respiratory illness. This reinforces the concern to take into account the route of exposure whenever assessing the health risks (remember the aforementioned case in Italy [47], where inhalation of aerosols contaminated with dinoflagellate cells occurred).

Some other routes of exposure were investigated for the toxicity assessment of PTX [49]. It was demonstrated that this substance is highly toxic after intramuscular or subcutaneous injection; no toxicity was found after intrarectal administration. Furthermore, PTX caused significant, non-lethal effects when topically applied to skin or eyes [38].

Returning to human health risks, by extrapolation of the animal toxicity data presented above, the toxic dose in a human was estimated to be between 2.3 and 31.5 μg PTX [12,43,50]. Recently, an acute reference dose was suggested to be 64 μg for a 60 kg individual [57].

## 3. Biological and Ecological Consequences of PTX and Its Analogs

A variety of marine toxins might accumulate in vectors that transfer it along food chains. As aforementioned, these may affect humans or other top predator organisms, leading in some cases to death. Not only adult individuals are affected. In fact, these toxins might also affect early life stages of invertebrate and vertebrate species [58]. Moreover, adults of some species may be insensitive to toxins while early stages are affected by them. In nature, the poisoning effects may be observed not only at the individual level, but also at the population level. If the impact is significant, the disturbance on a single ecologically important species may have repercussions in the whole ecosystem. In addition to the ecological repercussions, negative economic outcomes may also arise from the loss of the ecosystem value. These economic impacts can occur either by a decrease or disappearance of the population of a commercially valuable species in a given area or by a prohibition of seafood harvesting/fishing or consumption.

### 3.1. Effects in Invertebrate Larval Development

Not much is known about the effect of PTX and its analogs in invertebrates, and particularly in respect to developmental aspects. The few existing data are mainly related to ecological studies on the impact of *Ostreopsis* spp. in sea urchin communities and ecotoxicological effects in bivalves (see Section 3.3). The only documented effect of PTX in sea urchins reproduction is the inhibition of sperm motility [22]. Some other reported effects of PTX in invertebrates are retrieved from standard bioassays (Table 2). Toxicity caused by *Ostreopsis siamensis* isolates from New Zealand was studied for the brine shrimp *Artemia salina* and to larvae of the marine gastropod *Haliotis virginea* [59]. This dinoflagellate originating from New Zealand is known to produce PTX-like compounds [55]. *O. siamensis* killed brine shrimps, even at low cell numbers (250 cells per test well). The time until morbidity (tM_50_, when test organisms showed minimal gill movement) was 4 h, while the time until 50% death (LT_50_) was 24 h (Table 2). The dinoflagellate caused morbidity in gastropod larvae (tM_50_–1 h), but death did not occur within the 24 h of the bioassay, even at 1000 cells per test well. In this case, morbidity was characterized by retracted viscera, settled and velum lost.

### 3.2. Effects in Vertebrate Reproduction and Development

The motility of sperm from hamsters, guinea pigs, rabbits, cattle, humans and from the invertebrate sea urchins is inhibited by PTX exposure [22]. Its manifestation is characterized as a loss in flagellar-bend amplitude, which may be accompanied with an increase in beat frequency. The forward progression is lost gradually until the completely cessation of movement.

In animal developmental studies, the ability of PTX to block the Na^+^-K^+^ transporters and thus to depolarize the cell membrane is used. This capability was explored in such a study for *Xenopus laevis* [62], in which larvae were exposed to 2 nM PTX. It was shown that, at these concentrations, the larvae remain healthy and behave normally, although they do not regenerate their tails [62]. Some others potential physiological and developmental effects of PTX to vertebrates are also inferred from studies using the same model animal, e.g., by the frog embryo teratogenesis assay *Xenopus* (FETAX). By using this method, Franchini *et al*. [61] evaluated the toxicological effects of PTX in embryos at early gastrula stage of the anuran. It was found that at the highest toxin concentration tested (370 nM), the embryo population decreased by about 80% by the end of the assay (Table 2). An increase in the number of malformations and a delay in embryo growth were also observed. The modifications more frequently observed were folding along the antero-posterior body axis and swelling of the visceral mass. The histochemical analysis performed in the same study revealed the nature of the toxin-induced injuries. It was determined that the nervous system and the muscle tissue are sensitive target organs for PTX. Other relevant aspects include a general size reduction of main visceral organs, a severe damage to the heart structure and a negative inflammatory response.

These data highlight the putative morpho-functional changes that can be induced by exposure to PTX in vertebrates.

### 3.3. Toxins Distribution and Ecological Aspects

Toxin distribution, source organisms, and routes of exposure of PTX and its analogs are interrelated aspects that represent the main concern in respect to marine ecosystems and to human health issues. Since its discovery, PTX has been repeatedly documented in tropical Indo-Pacific seawaters, not only on the soft coral *Palythoa* spp. [3], but also in organisms associated to these zoanthid colonies [31]. Later, it was found that several species from the benthic dinoflagellates genus *Ostreopsis* also presents PTX-like compounds. Despite the uncertainty about the true origin for the production of these compounds discussed above (Section 1), *Palythoa* and *Ostreopsis* spp. are recognized as producers, and represent the major known sources for these toxins. In view of the fact that these two types of organisms were described from tropical and sub-tropical areas around the world, PTX and PTX-like compounds appeared to be confined to these biogeographic regions and thus largely overlooked. Nevertheless, possible new routes of exposure must be taken into account, such as the previously discussed cases of *Palythoa* corals present in aquariums. Moreover, *Ostreopsis* spp. have a broad geographical distribution than formerly thought [39], and are now a global concern since it can represent threats to human health whenever they develop and become prevalent [38]. Actually, while the presence of *Ostreopsis* spp. in tropical waters is well documented since the 1980s, the number of studies of these benthic dinoflagellates in temperate regions has increased substantially in the last few years [39]. These PTX-like producing microorganisms have been described for the China Sea [63], Pacific Ocean [55,63–65], Tasman Sea [65], Indic Ocean [9], Atlantic Ocean [50,66], Gulf of Mexico [67,68] and Mediterranean Sea [47,70–72,77]. The spread of toxin-producing *Ostreopsis* spp. to temperate regions may be due in part to ballast water of cargo ships and also to marginal changes in climate conditions, enough to induce bloom formation [39]. Dinoflagellate blooms are characterized by a brownish colored mucilaginous coverage of marine benthos.

Not surprisingly, due to the impact that these blooms may have, some studies [39,71] have been made in order to determine which are the optimal environmental conditions for the occurrence of this phenomena. Findings from these studies show that blooms are likely to be ephemeral and strongly related to seasonal patterns and to wave action [39,72]. The highest coverage occurs in hard substrata of shallow, sheltered areas such as harbors and estuaries [39,71], where the hydrodynamic conditions are gentle to moderate. In contrast to what occurs in other components of the microphytobenthos (e.g., diatoms, cyanobacteria), the mats formed are loosely attached to the substrata [71], and thus are easily resuspended in the water column by waves and mechanical action. This leads to cells dispersion, which may establish and develop in other areas whenever the conditions are favorable.

The high toxicity of PTX and its analogs has resulted several times in animal fatalities. Beside the few reports about human victims [38], animal deaths due to poisoning by PTX and analogs are also documented. These are the case of sea urchins poisoning after the occurrence of blooms of *Ostreopsis* spp., in Brazil and New Zealand [39,55,65], and the death of several pigs in Ryukyu Islands (Japan), after eating viscera of the filefish *Alutera scripta* [73]. In New Zealand, unexplained mouse deaths from regions where *Ostreopsis* spp. have been recorded raises the possibility that ostreocin-D or other PTX analog could have been the cause [59]. In Italy, massive mortalities of marine invertebrates and macroalgae [52], and visible impacts in sessile (cirripeds, bivalves, gastropods) and mobile (echinoderms, cephalopods, little fishes) epibenthos were also observed after summer blooms of *Ostreopsis ovata* [47,71].

Toxicological effects may arise directly from the toxin-producers itself (e.g., *Palythoa* spp. and *Ostreopsis* spp.) or indirectly via vectors that accumulate the toxin, which may be susceptible to the toxin or not. The entrance, diffusion and sequestration of PTX into the food chain have been recognized elsewhere [31]. For instance, in tropical and subtropical areas, PTX has been detected in several animals such as crabs [40], different species of fish [41,43] or organisms living in close association with *Palythoa* spp. The later include sponges, other soft corals, mussels, gorgonians and crustaceans or predators that feed on *Palythoa* spp. colonies such as the polychaete worm *Hermodice carunculata*, the starfish *Acanthaster planci* and the fish *Chaetodon* spp. [31]. These predators are examples of high tolerant organisms that store the toxin in its active form and may enable the distribution of PTX in other marine biota.

Filter-feeding invertebrates are also organisms in which PTX or analogs accumulation is a well known phenomenon, especially during harmful algal blooms [31,59,74,75]. Mussels, cockles, oysters, and scallops feed on toxic dinoflagellates, transferring them from the gills to digestive organs where the toxins accumulate [55,59]. PTX sequestration is also observed in sponges and mussels living near or among zoanthid colonies, which often exhibit higher PTX concentrations than *Palythoa* spp. [31]. It was showed that some shellfish are able to depurate the toxins at fairly rapid rates, while others can retain the toxins in its active form for months while continuing to bioaccumulate [31,76]. Moreover, the mode of accumulation can also differ in different bivalves. In an ecotoxicological study [55], the scallop *Pecten novaezealandiae* and the Pacific oyster *Crassostrea giga*s were fed with cells of a same culture of *Ostreopsis siamensis* (containing 0.3 pg palytoxin equivalents/cell), in numbers representative of a dense bloom. While the oysters contained detectable amounts of toxin in hepatopancreas, muscle, and roe, the scallops showed higher concentrations but only in the hepatopancreas. Unlikely these two shellfish, the green-lipped mussel *Perna canaliculus*, which was tested in the same study, did not present PTX-like substances in any of evaluated parts. Thus, it appears not to accumulate the toxin.

Notwithstanding the capability of shellfish to uptake PTX-like compounds after feeding on toxic *Ostreopsis* spp., no cases of human intoxications through consumption of contaminated shellfish have been noticed. The only suspected occurrence is a human shellfish poisoning that occurred with wild mussels collected in Tasmania [52]. As stated before, accumulated toxins may not be active. For instance, in the aforementioned Pacific oysters, no bioactivity was found by mouse bioassay [59], using extracts from individuals that fed on a sole diet of *O. siamensis* cells.

Concerning the effects that the toxin accumulation may have in such bivalves, little information is available. In one of the few exceptions [60,] it was found that PTX seriously increases phagocytosis (Table 2), a central process to the cell mediated immune response in invertebrates. The study was conducted on the mussel *Mytilus galloprovincialis*, a shellfish used for human consumption and widely used as a model. Nevertheless, it still lacks knowledge about the effects that this toxin can exert on mollusks in the long-term and in natural conditions.

Other marine organisms known to be affected by the toxins are sea urchins, an ecologically important herbivore. In southeastern Brazil, *Equinometra lucunter* individuals showed alterations on their exoskeleton, accompanied by high mortality. It did coincide with an outbreak of a benthic dinoflagellate, previously reported as belonging to *Prorocentrum* sp. but after confirmed to be *Ostreopsis ovata* [52]. Furthermore, algal extracts from this occurrence were used to carry out toxicity tests, namely the *Artemia salina* assay (Table 2). This marine invertebrate displayed 100% death after being exposed to an extract of no more than 125 *O. ovata* cells [66]. Chemical analyses confirmed the presence of a PTX-like compound in samples. A further toxicological test with *O. ovata* extracts from the Mediterranean Sea revealed a similar association between *Artemia salina* mortality after 24 h and the number of algal cells [52,77]. In this case, 542 to 906 cells/mL were sufficient to cause 65% to 100% death, respectively. Recently, it was also observed that summer blooms of *O. siamensis* killed sea urchins in New Zealand [57]. Another study from this austral country corroborates these findings [39]. A strong negative effect between the health of the sea urchin *Evechinus chloroticus* and the benthic cover of *O. siamensis* was demonstrated. The echinoderm densities declined by 56–60% at bloom sites over the study period (Table 2).

## 4. Summary

PTX and its analogs are potent marine toxins known to cause fatality of several animals, including humans. Even so, despite this obvious acute biological impact, little is known about the real consequences that these toxins may have on coastal communities. Because of the human health risks, attempts continue to be made aiming for the development of a validated assay for the rapid, sensitive and specific detection of PTX and/or analogs. The accomplishment of these requisites and its optimization have been evaluated or developed in some of the aforementioned studies and comprise either analytical methods or biological assays. Nevertheless, none are able to meet all the requirements *per se*, and so a combination of fast and confirmatory methods still seems to be the more appropriate approach for monitoring purposes. Moreover, it is also expected that once the biochemistry and molecular genetics involved in the biosynthesis of these toxins is elucidated, new methodological approaches will be possible for the detection of PTX and PTX-like producers.

Marine organisms associated with soft corals in tropical and subtropical regions appear to be adapted to the presence of PTX. Respecting to temperate climates, where the biota are supposed to be more susceptible to the toxin, the impact is quite obvious. Data on the biological and ecological effects of these toxins presented previously expose these findings. They show that different organisms, belonging to diverse trophic levels of the marine food chain, are susceptible to being affected by these toxins, some of them commercially valuable seafood products. Nevertheless, the data are retrieved mainly from *ex situ* toxicological studies and from a few reports about community structure changes observed in natural populations. This information is an important contribution to better understand the effect that these toxins may have in marine food webs and ecosystem structure and function. However, the true ecological impact of the distribution of PTX and its analogs still needs to be assessed. It will be relevant that future studies highlight the impact of the toxins along horizontal and vertical levels of the food chain, in different ecosystems, and to the mid- and long-term. Moreover, it will be important to survey the dynamics of the expansion of these toxins worldwide, in particular by monitoring other temperate regions.

## Figures and Tables

**Table 1:** Toxicity reference values of PTX and its analogs administered by several different routes on two model animals.

Test animal	Toxin type	Toxin source	Route of exposure	24 h LD_50_ (μg/kg)	References
Rat	Palytoxin	semi-purified material from *Palythoa* spp.	intravenous	0.089	[38,49]
	“ “	“ “	subcutaneous	0.4	[38,49]
	“ “	“ “	intragastric	>40.0	[38,49]
	“ “	“ “	intraperitoneal	0.63	[38,49]
	“ “	“ “	intratracheal	0.36	[38,49]
Mouse	Palytoxin	“ “	intravenous	0.045	[38,49]
	“ “	“ “	subcutaneous	1.39	[38,49]
	“ “	*P. caribaeorum*	oral	510	[29]
	“ “	*P. tuberculosa*	“ “	767	[53]
	“ “	“ “	intraperitoneal	0.295	[51]
	Ostreocin-D	*Ostreopsis siamensis*	“ “	0.75	[7,52]
	Mascarenotoxin-A	*O. mascarenensis*	“ “	900	[9,12]
	Ostreotoxin *	*O. lenticularis*	“ “	32100	[12,54]
N.T.	Ovatoxin-A	*O. ovata*	-	-	[11]

N.T.—not tested;

* —classification as palytoxin analog not yet established.

**Table 2:** Effects of PTX or analogs on the survival of several animal species.

Animal species	Toxin type/Producer	Concentrations tested or cell densities	Toxicity/Observed effects	Refs
**Invertebrates**				
*Haliotis virginea* (sea snail)	Ostreocin-D/*Ostreopsis siamensis*	1000 cells per test well	tM_50_: 1 h not lethal within 24 h	[59]
*Artemia salina* (brine shrimp)	“ “	250 cells per test well	tM_50_: 4 h TL_50_: 24 h	[59]
“ “	Ovatoxin-a?/*O. ovata*	125 cells per test well	100% mortality	[52]
“ “	“ “	542 – 906 cells mL^−1^	65%–100%, in 24 h	[52,77]
*Equinometra lucunter* (rock boring urchin)	“ “	Algal bloom outbreak	Exoskeleton changes High mortality	[52]
*Evechinus chloroticus* (New Zealand sea urchin)	“ “	Algal bloom outbreak	density ↓ 56–60%	[39]
*Pecten novaezealandiae* (New Zealand scallop)	Ostreocin-D/*O. siamensis*	0.3 pg PTX equivalents cell^−1^	No toxic effects Toxin accumulation	[55]
*Crassostrea gigas* (Pacific oyster)	“ “	“ “	“ “	[55]
*Perna canaliculus* (green-lipped mussel)	“ “	“ “	No toxic effects No tissue accumulation	[55]
*Mytilus galloprovincialis* (Mediterranean mussel)	Palytoxin/*Palythoa* sp.?	2 ng PTX mL^−1^ 20 ng PTX mL^−1^	strongly ↓ phagocytosis lysis of >30% of cells	[60]
**Vertebrates**				
*Xenopus laevis* (African clawed frog)	Palytoxin/*Palythoa* sp.	370 nM PTX	TL _50_: 3 days *	[61]

* —values are calculated from data reported by authors;

TL_50_—lethal time for 50% of the tested organisms;

tM_50_—time until morbidity for 50% of the tested organisms.
